# Multiple roles of apolipoprotein B mRNA editing enzyme catalytic subunit 3B (APOBEC3B) in human tumors: a pan-cancer analysis

**DOI:** 10.1186/s12859-022-04862-0

**Published:** 2022-08-02

**Authors:** Jiacheng Wu, Ni Li, Linwen Zhu, Dawei Zhen, Mengqi Li, Hang Chen, Mengmeng Ye, Yiqin Wei, Guofeng Shao

**Affiliations:** 1 Medical College, Ningbo University, Ningbo, Zhejiang People’s Republic of China; 2 Department of Cardiothoracic Surgery, Lihuili Hospital Affiliated to Ningbo University, Ningbo, 315000 Zhejiang People’s Republic of China; 3 Department of Geriatrics, Shaoxing People’s Hospital (Shaoxing Hospital, Zhejiang University School of Medicine), Shaoxing, Zhejiang People’s Republic of China

**Keywords:** *APOBEC3B*, Oncogenic role, Pan-cancer analysis

## Abstract

Although there have been some recent cell and animal experiments indicating that expression of the gene encoding apolipoprotein B mRNA editing enzyme catalytic subunit 3B (*APOBEC3B*) is closely related to cancer, it still lacks pan-cancer analysis. Here we analyzed the potential carcinogenic role of *APOBEC3B* in 33 tumors based on The Cancer Genome Atlas (TCGA). *APOBEC3B* was highly expressed in most tumors and weakly expressed in a few. Differences in expression level were significantly correlated with the pathological tumor stage and prognosis of affected patients. The high-frequency *APOBEC3B* changes were principally mutations and amplifications in some tumors, such as uterine corpus endometrial carcinomas or cutaneous melanomas. In testicular germ cell tumors and invasive breast carcinomas, *APOBEC3B* expression and CD8^+^ T lymphocyte counts were correlated. In other cancers, such as human papilloma virus (HPV)-related head and neck squamous cell carcinomas or esophageal adenocarcinomas, there was also cancer-associated fibroblast infiltration. The *APOBEC3B* enzyme acts in the mitochondrial respiratory electron transport chain and in oxidative phosphorylation. This first pan-cancer study provides a comprehensive understanding of the multiple roles of *APOBEC3B* in different tumor types.

**Supplementary Information:**

The online version contains supplementary material available at 10.1186/s12859-022-04862-0.

## Introduction

Over the past decades, the incidence of cancer has increased rapidly. Cancers are among the leading causes of death worldwide, so the therapeutic success rate needs to be further improved [1, 2]. In this regard, the mechanisms of tumorigenesis and progression are complicated and deserve further investigation. Pan‐cancer analysis has emerged as a widely used method to investigate the common features and heterogeneities of various tumor types. It allows for a thorough and profound understanding of human cancers [3]. The publicly funded The Cancer Genome Atlas (TCGA) project contain functional genomics datasets for different tumors, [4, 5] which enable pan-cancer analysis.

The apolipoprotein B mRNA editing enzyme catalytic subunit 3B (*APOBEC3B*) protein, also known as A3B or ARP4, is a member of the cytidine deaminase gene family [6]. It is one of seven related genes or pseudogenes found in a cluster on human chromosome 22, thought to result from gene duplication. Members of the cluster encode proteins that are structurally and functionally related to the C-to-U RNA-editing cytidine deaminase APOBEC [7–9]. *APOBEC3B* is involved in the development and progression of breast cancers [10], hepatocellular carcinomas [11], ovarian cancers, [12] nasopharyngeal carcinomas, [13] and chondrosarcomas [14]. Comprehensive analysis of the expression patterns, prognostic significance and relationship with tumor microenvironment of *APOBEC3B* at pan-cancer level will help us deepen our understanding of this enzyme.

Here, we conducted a pan-cancer analysis of *APOBEC3B* using the TCGA project. We also analyzed the pathogenesis or potential molecular mechanisms of *APOBEC3B* in different cancers by gene expression, patient survival status, protein expression, gene mutation, immune infiltration, protein interactions and cellular pathways. These findings could strengthen our understanding of the biological functions of *APOBEC3B* in different cancer types.

## Materials and methods

### Gene expression analysis

We entered *APOBEC3B* into the ‘GENE_DE’ module of ‘Exploration’ on the Tumor Immune Estimation Resource (TIMER2, v. 2) web page (http://timer.cistrome.org/) [15–17] and observed the differential expression of *AOPBEC3B* in different tumors and adjacent normal tissues in the TCGA project. For certain tumors that lacked normal adjacent tissue samples (e.g., TCGA-ACC for adrenocortical carcinomas; TCGA-OV for ovarian serous cystadenocarcinomas), we used the UCSC XENA database (https://xenabrowser.net/datapages/) [18] downloaded the TCGA and the Genotype-Tissue Expression (GTEx) of transcripts per million reads (TPM) RNAseq data in the format processed uniformly by the Toil process [19]. Then we performed log_2_ transformation of these data, compared the expression between samples of tumor tissues and corresponding normal tissues, and have presented the results in box plots. Gene expression differences were visualized with the ‘ggplot2’ R package. The R language software (R-3.6.3, 64 bit; http://www.r-project.org/) was used in this analysis. In addition, we used the ‘Expression DIY-Pathological Stage Plot’ of the Gene Expression Profiling Interactive Analysis, web services (GEPIA2 v. 2; http://gepia2.cancer-pku.cn/analysis) [20] to obtain violin plots of *APOBEC3B* expression levels in different pathological stages (stages I through IV) of all TCGA tumors. All the above box plots or violin plots were based on log_2_(TPM + 1) transformed expression data.

We performed protein expression analysis on the Clinical Proteomic Tumor Analysis Consortium (CPTAC) dataset using the UALCAN web resource (http://ualcan.path.uab.edu/index.html), [21] a comprehensive, interactive web for analyzing cancer omics data. We entered ‘*APOBEC3B*’ to analyze differences in *APOBEC3B* total protein levels between tumors and adjacent normal tissues. The available data sets for eight tumor types were selected: breast cancers, ovarian cancers, renal clear cell carcinomas (RCC), uterine corpus endometrial carcinomas (UCEC), lung adenocarcinomas (LUAD), head and neck squamous carcinomas (HNSC), pancreatic adenocarcinomas (PAAD), and liver hepatocellular carcinomas (LIHC).

### Survival analysis

We used the ‘Survival Analysis-Survival Map’ module of GEPIA2 to obtain the survival heat map of overall survival (OS) and disease-free survival (DFS) of *APOBEC3B* for patients with all TCGA tumors, estimated using the Mantel–Cox test. We used months as the survival time unit, high cutoff (50%) and low cutoff (50%) values as the segmentation criteria of high expression and low expression cohorts and *P <* 0.05 as the measure of significance level. Subsequently, we analyzed tumors with significant differences in patient survival in heat maps through the ‘survival analysis’ module of GEPIA2 and obtained the corresponding survival plots.

### Gene alteration analysis

We selected ‘TCGA pancancer atlas studies’ in the ‘quick select’ option on the home page of the cBioPortal website (https://www.cbioportal.org/), [22] and then entered ‘*APOBEC3B*’ to query its gene alterations. In the ‘Cancer Type Summary’ module, the alteration frequency, mutation type and copy number alteration (CNA) results of all TCGA tumors were observed. In the ‘Mutations’ module, the mutation site information of *APOBEC3B* is displayed in the protein structure track or three-dimensional (3D) structure diagrams. After that, we analyzed the overall, disease-specific, disease-free and progression-free data of TCGA cancer cases with *APOBEC3B* gene alterations through the ‘Comparison/Survival’ module, and obtained the corresponding Kaplan–Meier plots with log-rank test P-values.

### Immune infiltration analysis

We used the ‘Immune gene’ module of the TIMER2 website to evaluate the correlation between *APOBEC3B* and immune infiltration expression in all TCGA tumors, entered ‘*APOBEC3B*’ in gene expression and selected ‘T-cells CD8 + ’ and ‘cancer-associated fibroblasts’ in the immune information menu. Then, the immune infiltration data were analyzed by TIMER, EPIC, MCPCOUNTER, XELL, TIDE, CIBERSORT, CIBERSORT-ABS algorithms, and then the P-values and partial correlation values were obtained using a purity-adjusted Spearman’s rank correlation test. Finally, heatmaps and scatter diagrams were obtained after the data were visualized.

### APOBEC3B-related gene enrichment analysis

After logging into the STRING (https://cn.string-db.org/) website, [23] we first entered the protein name ‘*APOBEC3B*’ and selected the organism as ‘*Homo sapiens*’, Then we set the parameters as follows: meaning of network edges (‘Confidence’), active interaction sources (‘Textmining’, ‘Experiments’, ‘Databases’, ‘Co-expression’, ‘Gene Fusion’, and ‘Co-occurrence’), minimum required interaction score (‘low confidence 0.150’) and maximum number of interactors to show ‘no more than 50 interactors’. Ultimately, we obtained the top 50 *APOBEC3B*-related evidence-based proteins and corresponding network diagrams.

To search for genes with an expression pattern similar to *APOBEC3B* in different cancer types, we entered the gene ‘*APOBEC3B*’ and ‘top 100’ in the ‘Expression Analysis-Similar Gene Detection’ module of the GEPIA2 website, and selected all TCGA tumor data sets for analysis. Subsequently, we selected the top five genes from the list, entered the gene’*APOBEC3B*’ and ‘top5 genes’ in the ‘expression analysis correlation analysis’ module of GEPIA2, selected the Pearson correlation coefficient, and performed a visual correlation analysis on the entire TCGA tumor dataset. The dot plots obtained were scaled by log_2_ TPM and contained the P-values and the correlation coefficients (R values). In addition, we used the ‘Exploration Gene_Corr’ module of TIMER2 to obtain the heatmap data related to the top five genes and *APOBEC3B* in various cancer types, which gave us the purity-adjusted partial Spearman’s rho value as the degree of their correlation.

We used the ‘Draw Venn Diagram’ website (http://bioinformatics.psb.ugent.be/webtools/Venn/) to cross-analyze the previously acquired ‘Top50 gene’ of STRING and ‘Top100 gene’ data from GEPIA2. Subsequently, we performed Kyoto Encyclopedia of Genes and Genomes (KEGG) pathway analysis for the above genes. We entered the gene list on the David (https://david.ncifcrf.gov/) [24] website and selected ‘OFFICAL_GENE_SYMBOL’ and ‘*Homo sapiens*’ to finally obtain functional annotation data. In addition, we used the ‘clusterprofiler’ (v. 3.14.3) for enrichment analysis and the ‘org.Hs.eg.db’ (v. 3.10.0 for ID transformation) R packages to perform gene ontology (GO) enrichment analysis. The resulting KEGG and GO analysis including biological process (BP), cellular component (CC), and molecular function (MF) data described above were visualized as bubble plots and network diagrams via the ‘ggplot2’ and ‘clusterprofiler’ R packages (v. 3.3.3). All R languages in this article are based on the R software (R-3.6.3, 64bit; http://www.r-project.org/).

## Results

### Expression analysis of APOBEC3B

In this study, we principally explored the potential role that *APOBEC3B* (NM_004900.4 for the mRNA or NP_004891.4 for the protein; Fig. S1a) might play during cancer initiation and progression. The gene encoding *APOBEC3B* is conserved in the chimpanzee, dog, cow, mouse, and rat, and 20 organisms have orthologs with human *APOBEC3B*. [25] We investigated the expression profile of the *APOBEC3B* gene in cells from different normal tissues, as shown in Additional file 2: Figure S1b, the mRNA expression of *APOBEC3B* showed the highest level in bone marrow, followed by the appendix and colon, based on the Human Protein Atlas dataset.

**Fig. 1:**
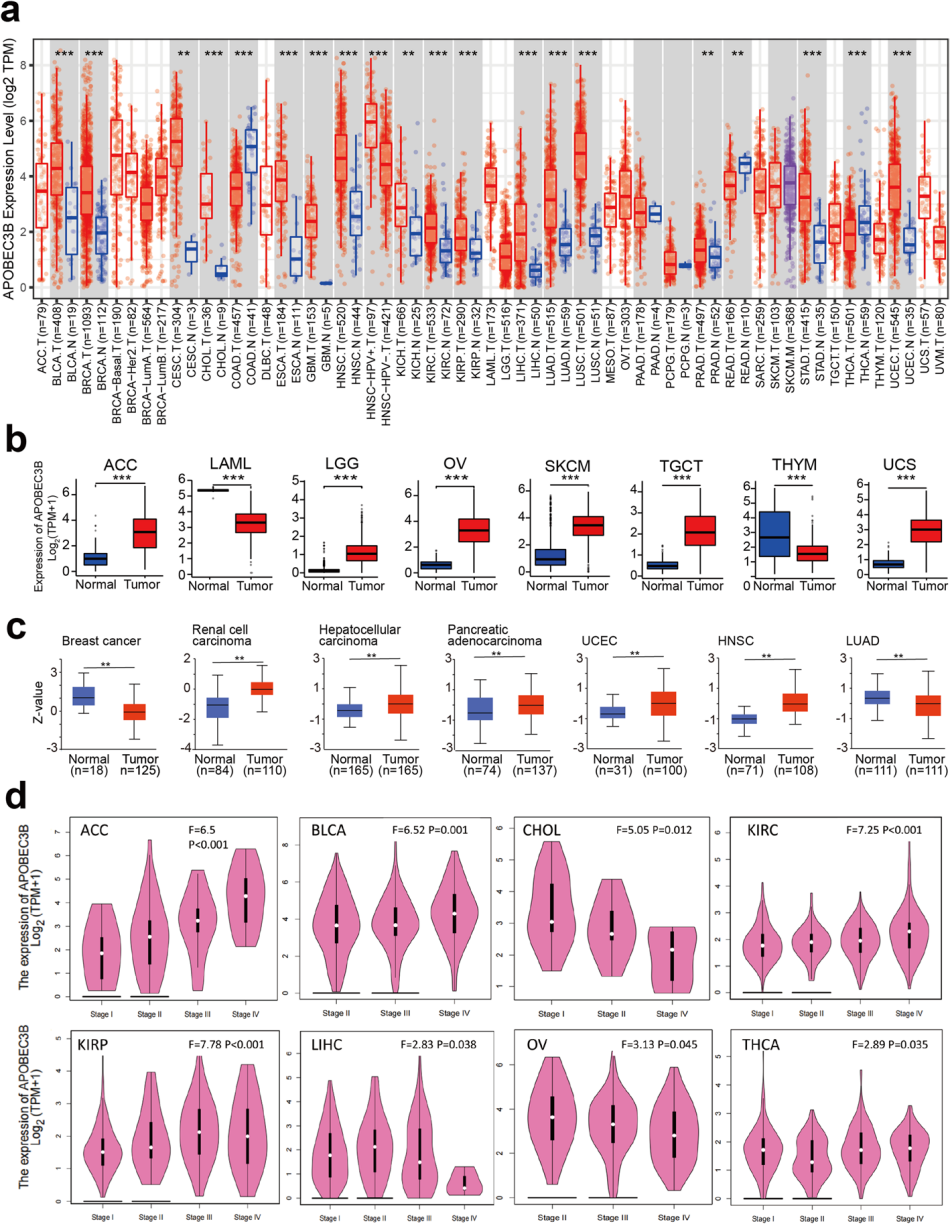
Expression level of the *APOBEC3B* gene in different tumors and pathological stages. **a** The mRNA expression of the *APOBEC3B* in different tumor subtypes and adjacent normal tissues. **P < 0.01; ***P < 0.001. **b** The mRNA expression of the *APOBEC3B* in ACC, LAML, LGG, OV, SKCM, TGCT, THYM, and UCS tumor types in the TCGA dataset, corresponding normal tissues of the GTEx database were included as controls. ***P < 0.001. **c** The protein levels of *APOBEC3B* between normal tissues and primary tissues of breast cancers, clear cell RCC, hepatocellular carcinomas, PAAD, UCEC, HNSC, and LUAD, based on the CPTAC dataset. **P < 0.01. **d** The relationship between *APOBEC3B* expression and clinical stages in ACC, BLCA, CHOL, KIRC, KIRP, LIHC, OV, and THCA tumor types

We analyzed *APOBEC3B* expression levels in different cancer types from TCGA using the TIMER2 website. As shown in Fig. 1a, the expression level of *APOBEC3B* was higher in the tumor tissues of bladder urothelial carcinomas (BLCA), breast invasive carcinomas (BRCA), cholangiocarcinomas (CHOL), esophageal carcinomas (ESCA), glioblastoma multiforme (GBM), HNSC, kidney renal clear cell carcinomas (KIRC), kidney renal papillary cell carcinomas (KIRP), LIHC, LUAD, lung squamous cell carcinomas (LUSC), stomach adenocarcinomas (STAD), UCEC (*P <* 0.001), cervical squamous cell carcinomas and endocervical adenocarcinomas (CESC), kidney chromophobes (KICH), prostate adenocarcinomas (PRAD) (*P <* 0.01) than in the corresponding normal tissues. However, it was lower in the tumor tissues of colon adenocarcinomas (COAD), thyroid carcinomas (THCA) (*P <* 0.001) and PRAD (*P <* 0.01).

For those TCGA tumors with missing normal tissue data, we acquired the normal tissue data of the GTEX dataset and used them as a control group to further analyze the expression differences of *APOBEC3B* in normal tissues and tumor tissues of Adrenocortical carcinoma (ACC), acute myeloid leukemia (LAML), OV, testicular germ cell tumors (TGCT), uterine carcinosarcomas (UCS), brain lower grade gliomas (LGG), skin cutaneous melanomas (SKCM) and thymomas (THYM) (Fig. 1b , *P* < 0.001). Among them, LAML and THYM were weakly expressed in tumor tissues. Nevertheless, we did not find significant differences in the expression of *APOBEC3B* in lymphoid neoplasm diffuse large B-cell lymphomas (DBLC), mesotheliomas (MESO), pheochromocytomas and paragangliomas (PCPG), or uveal melanomas (UVM) tissues.

To further explore the proteomics data of *APOBEC3B* in patients with cancers, we conducted an analysis using the CPTAC dataset. The *APOBEC3B* protein expression level was higher in renal clear cell carcinomas, UCEC, HNSC, PAAD and LIHC and lower in breast cancers and LUAD (Fig. 1c, all *P <* 0.01) than in normal tissues. Interestingly, the proteomics data suggested lower expression of the *APOBEC3B* protein in breast cancer and LUAD, opposite of that observed at the mRNA level. These inconsistent results might be caused by post-transcriptional or translational regulation.

In addition, we used the ‘Pathological Stage Plot’ module of GEPIA2 to analyze the relationship between the expression of *APOBEC3B* and the pathological stages of cancers. As shown in Fig. 1d, we obtained the pathological stage plots of ACC, BLCA, CHOL, KIRC, KIRP, LIHV, OV and THCA (all *P <* 0.05). To be specific, in ACC, BLCA, KIRC, and KIRP tissues, *APOBEC3B* RNA expression levels were consistently associated with later clinical stages; in CHOL, LIHC, and OV mRNA expression level of *APOBEC3B* was significantly higher in the early stage of these diseases.

Thus, *APOBEC3B* has different expression levels in different tumor tissues; the expression levels were closely related to the pathological state of the tumor, but the correlation was not consistent across different cancer species. These findings indicated a diverse biological function of this enzyme in different tumor types.

### Survival analysis data

**Fig. 2:**
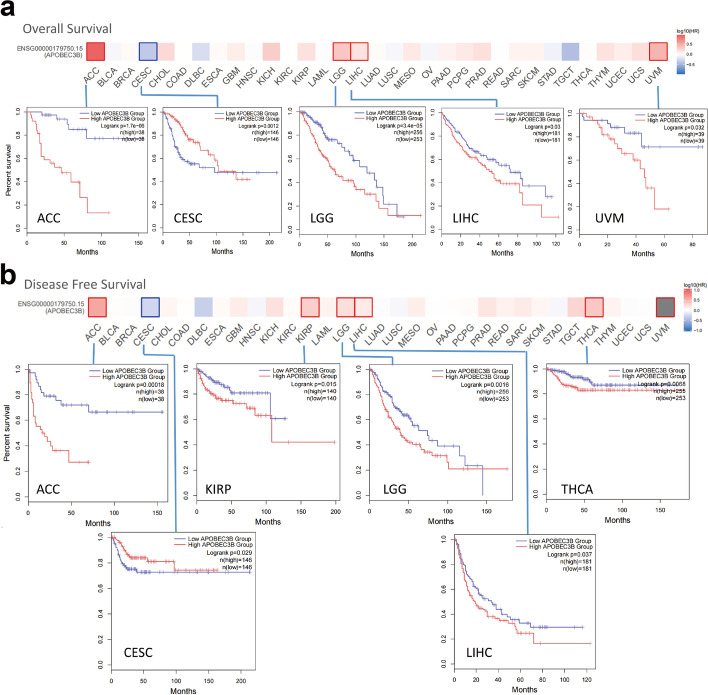
Correlation between *APOBEC3B* gene expression and survival prognosis for patients with cancers in TCGA. We performed overall survival (OS) (**a**) and disease-free survival (DFS) (**b**) analyses of different tumors in TCGA according to *APOBEC3B* gene expression. The survival maps and Kaplan–Meier curves with positive results were provided

Taking the median expression level of *APOBEC3B* in tumor cases as the standard, we divided the tumor cases in TCGA datasets into high- and low-expression groups, and explored the relationship between the expression of *APOBEC3B* and the prognoses for different patients. As shown in Fig. 2a, the tumors associated with high expression of *APOBEC3B* and poor OS were ACC (*P <* 0.001), LGG (*P <* 0.001), LIHC (*P = *0.03) and UVM (*P = *0.032) within the TCGA project In the DFS analysis (Fig. 2b). A high expression level of *APOBEC3B* was associated with poor prognosis for patients with ACC (*P <* 0.001), KIRP (*P = *0.015), LGG (*P = *0.0016), LIHC (*P = *0.037), and THCA (*P = *0.0068). Furthermore, low expression of *APOBEC3B* was related to poor OS and DFS prognoses for patients with CESC (Fig. 2a , *P* = 0.0012; Fig. 2b , *P* = 0.029). These inconsistent results in different cancer species suggested that *APOBEC3B* might play different roles in different tumor types.

We also analyzed the clinical prognostic significance of DNA methylation of *APOBEC3B* in the website tool (https://biit.cs.ut.ee/methsurv/). We have listed the results in Additional file 1: Table S1. Methylation at cg06837067 had the most prognostic significance, predicting a poor overall survival in ACC (HR: 2.179, *P = *0.04), BLCA (HR: 1.924, *P <* 0.01), CESC (HR: 2.034, *P <* 0.01), HNSC (HR: 1.556, *P = *0.01), KIRC (HR: 2.353, *P <* 0.01), KIRP (HR: 2.258, *P = *0.01), SKCM (HR: 1.735, *P <* 0.01), and STAD (HR: 1.526, *P <* 0.01), however, in LGG (HR: 0.343, *P <* 0.01), MESO (HR: 0.564, *P = *0.045), and UVM (HR: 0.229, *P = *0.046), the methylation level at cg06837067 predicted greater overall survival (Additional file 1: Table S1). This further suggested that *APOBEC3B* might play different roles in different cancer types.

### Gene alteration analysis

**Fig. 3:**
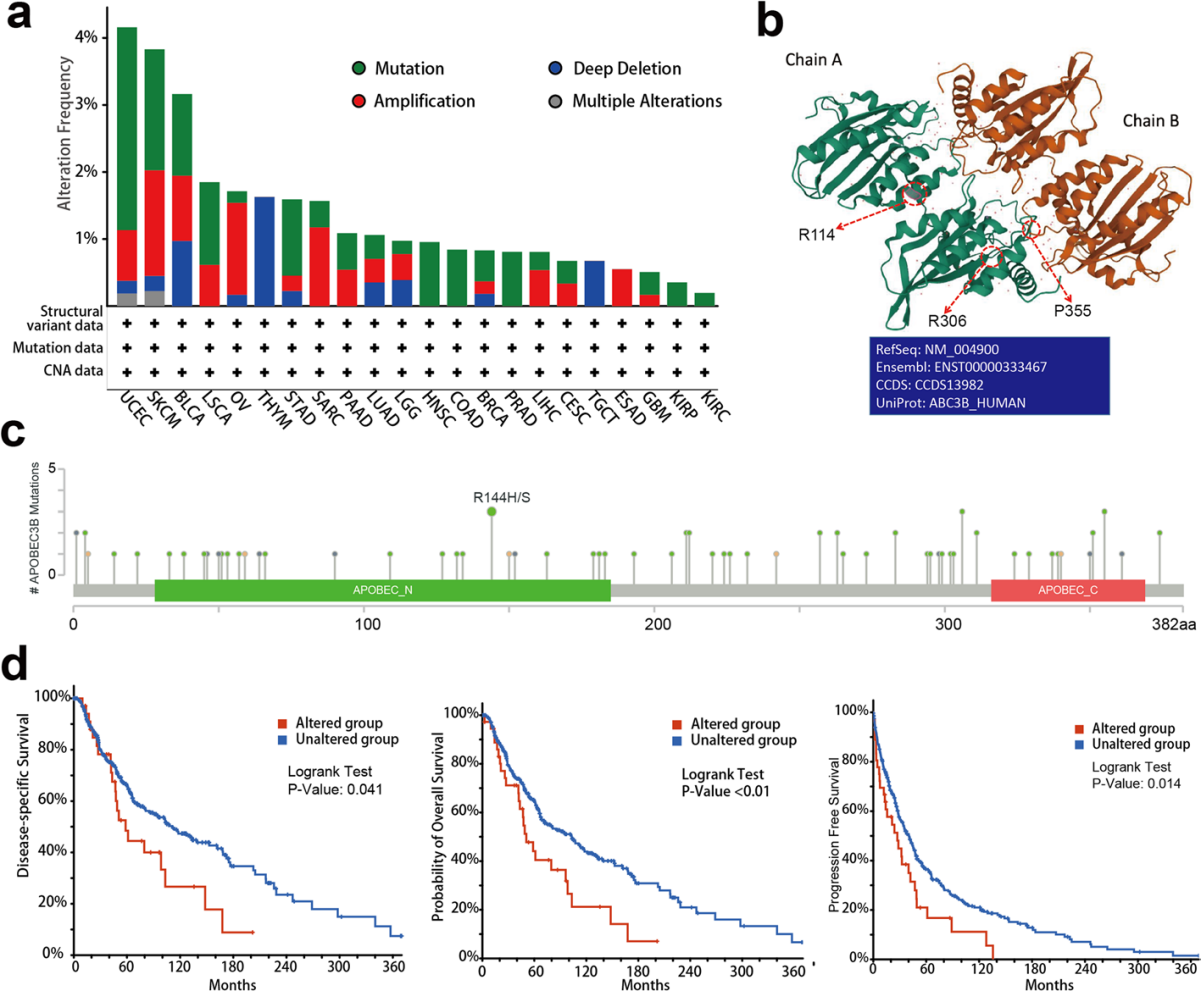
Mutation feature of *APOBEC3B* in different tumors listed in TCGA. **a, b** The mutation features of *APOBEC3B* for the TCGA-listed tumors using the cBioPortal tool. The alteration frequencies with mutation type (**a**) and mutation site (**b**) were displayed. **c** The highest alteration frequency of mutation sites (R114, R306, R355) of *APOBEC3B* were shown in the 3D structure. **d** The potential correlation between mutation status and disease-specific, overall and progression-free survival in patients with SKCM

We analyzed the gene alteration status of *APOBEC3B* in all tumor samples in the TCGA dataset through cBioPortal. As shown in Fig. 3a, the tumor type with the highest alteration frequency of *APOBEC3B* was UCEC (> 4%), mainly of the ‘mutation’ type. The second most frequent alteration was in SKCM (~ 3.7% frequency). In cases with ovarian serous cystadenocarcinomas, the main type was ‘amplification’, and the alteration frequency was ~ 2%. It is worth mentioning that all types of genetic alteration (~ 2% frequency) in cases of thymomas were of the ‘deep deletion’ type. In addition, the types, loci and corresponding cases of *APOBEC3B* gene alteration are shown in Fig. 3c. Missense mutations were found to be the main type of *APOBEC3B* genetic alterations: an R114H/S alteration in the APOBEC-like N-terminal domain, which was detected in one case of GMB, one case of PRAD and one case of STAD (Fig. 3b), and a P355S alteration in APOBEC-like C-terminal domain, which was detected in one case of LUSD and two cases of SKCM. These alterations are able to induce a frame shift mutation of the *APOBEC3B* gene, from R (Arginine) to H (Histidine) or S (Serine) at site 114 and P (Proline) to S at site 355 of the *APOBEC3B* protein, and subsequent *APOBEC3B* protein truncation. We also noted the sites of R144,R306 and P335 in the 3D structure of *APOBEC3B* protein (Fig. 3c). In addition, we also analyzed the association between *APOBEC3B* gene alteration and clinical survival and prognosis with different types of cancers. As shown in Fig. 3d, the *APOBEC3B* altered group in patients with SKCM showed better prognosis in DSS (*P = *0.0411), OS (*P <* 0.01) and progression-free (*P = *0.0137) survival, but in UCEC cases overall, disease-specific, disease-free and progression-free patient survival rates were not statistically significant.

### Immune infiltration analysis data

**Fig. 4:**
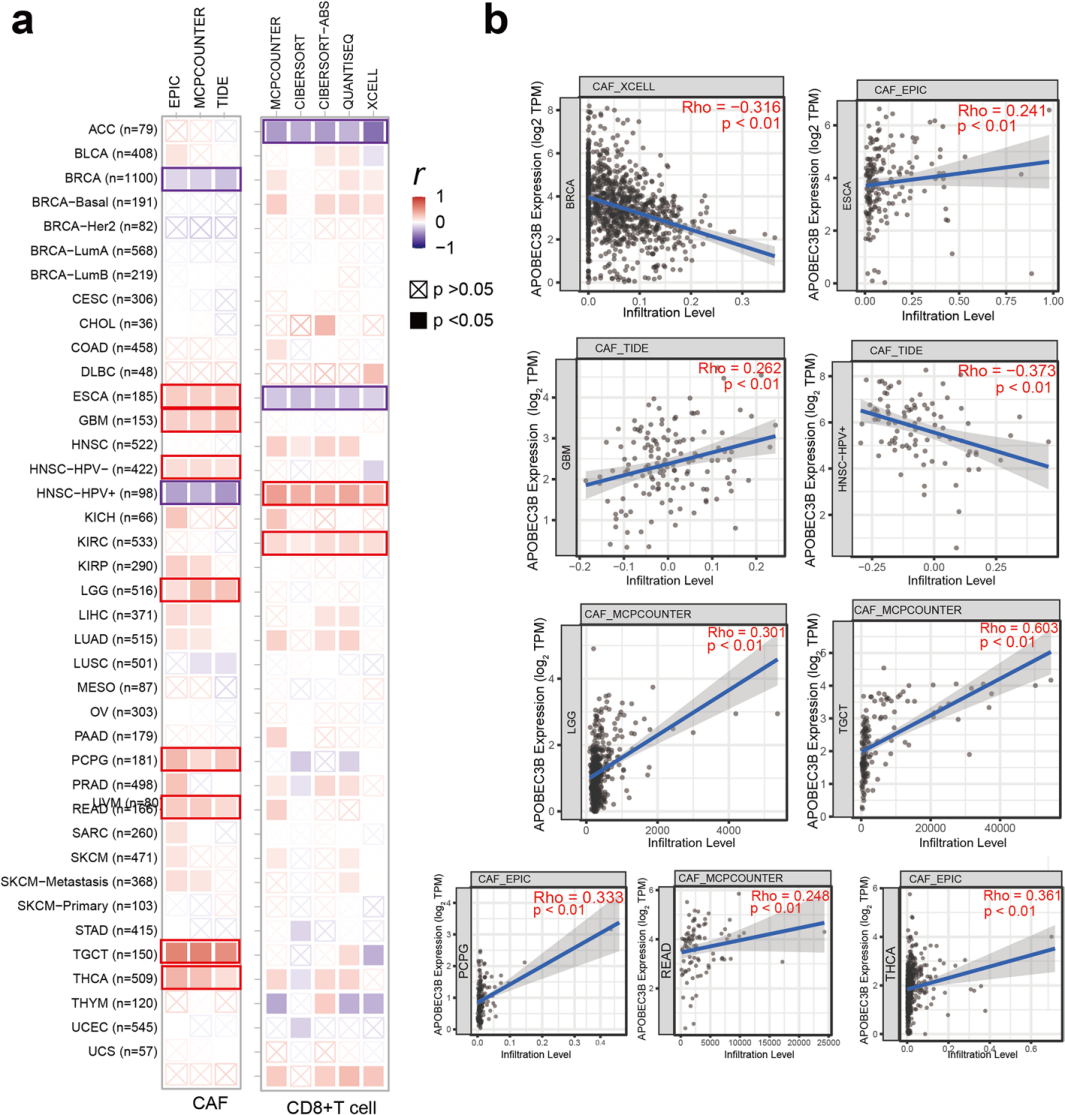
Correlation analysis between *APOBEC3B* expression and immune infiltration of cancer-associated fibroblasts and CD8^+^ T-cells. **a** Heatmap showing the correlation between the expression levels of the *APOBEC3B* and the infiltration level of cancer-associated fibroblasts/CD8^+^ T-cells obtained by different algorithms. **b** The correlation between *APOBEC3B* expression and immune infiltration cancer-associated fibroblasts in some TCGA-listed tumors

**Fig. 5:**
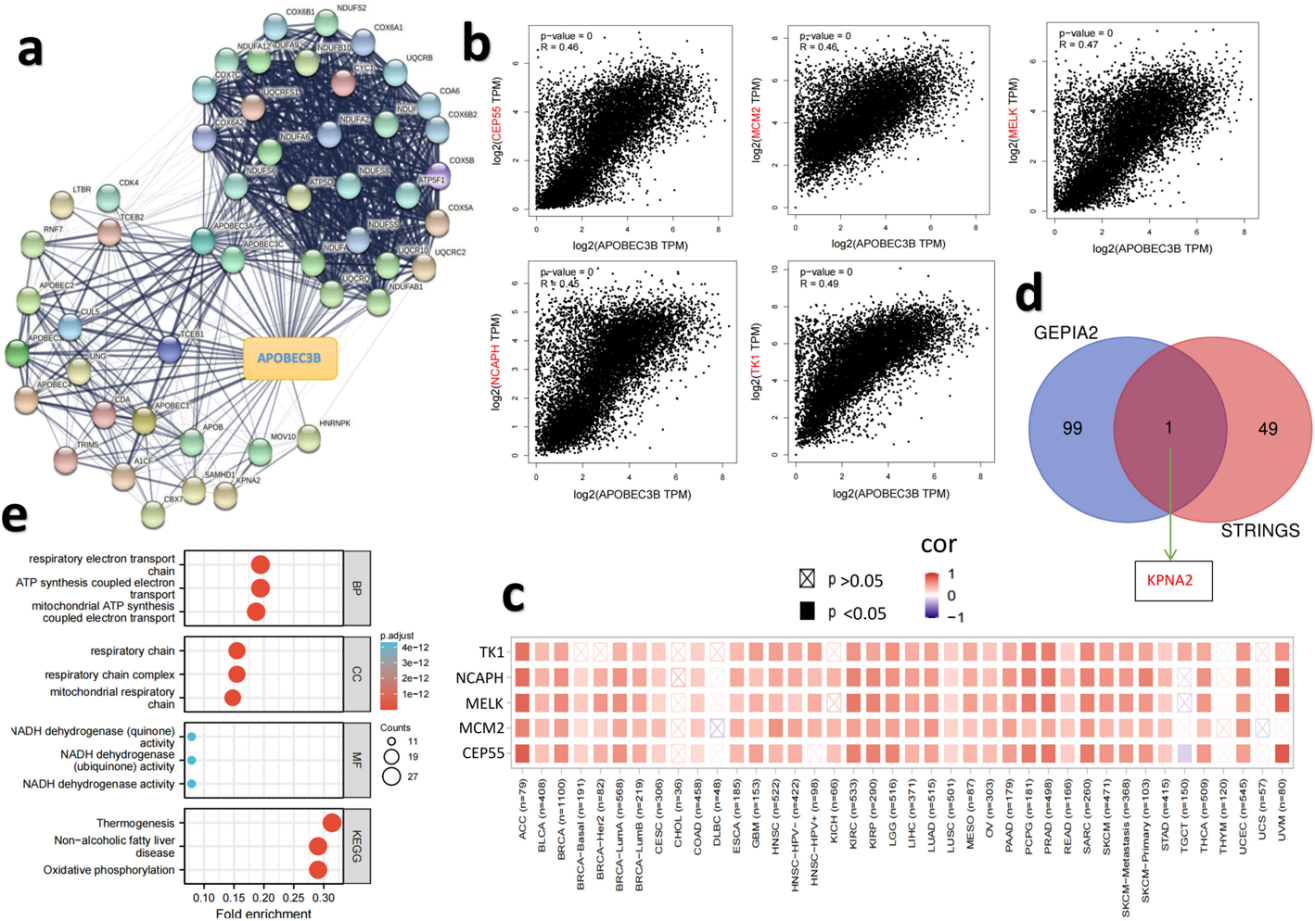
*APOBEC3B*-related gene enrichment analysis. **a** The *APOBEC3B*-binding proteins identified using the STRING tool. **b** The correlation between the expression of *APOBEC3B* and top 5 genes co-expression with *APOBEC3B* (TK1, MELK, CEP55, MCM2, and NCAPH). **c** Heatmap showing the correlation between the expression of *APOBEC3B* and top 5 genes co-expression with *APOBEC3B* (TK1, MELK, CEP55, MCM2, and NCAPH) in the detailed cancer types. **d** Venn diagram showing the intersection analysis of the *APOBEC3B*-binding and correlated genes. **e** Bubble chart of KEGG pathway analysis and GO enrichment analysis based on the *APOBEC3B*-binding and interacting genes

Tumor-infiltrating immune cells play an important role in the occurrence, progression and metastasis of tumors [26]. We used the TIMER, EPIC, MCPCOUNTER, XELL, TIDE, CIBERSORT, and CIBERSORT-ABS algorithms to explore correlations between the infiltration level of various immune cells and the expression level of *APOBEC3B* gene in different TCGA tumors. Cancer-associated fibroblasts are the main component of the stroma and secrete growth factors, inflammatory ligands and extracellular matrix proteins to promote tumor proliferation, therapeutic resistance and immune rejection. After a series of analyses, we observed that the infiltration value of cancer-associated fibroblasts in ESCA, GBM, HNSC-HPV^−^, LGG, PCPG, rectum adenocarcinoma (READ), TGCT, and THCA type tumors were positively correlated with the expression level of *APOBEC3B*, but negatively correlated with BRCA and HNSC-HPV^+^ tumor types (Fig. 4a). Moreover, we observed a statistically significant positive correlation between the immune infiltration of CD8^+^ T-cells and the expression level of *APOBEC3B* in HNSC0-HPV^+^ and UVM tumors based on most algorithms, while the ESCA tumor type was negatively correlated (Fig. 4b). Finally, as shown in Fig. 5c, we selected an algorithm to obtain a scatterplot of this tumor. For example, based on XCELL algorithm, the expression level of *APOBEC3B* in BRCA was negatively correlated with the infiltration level of cancer-associated fibroblasts (Fig. 5c , *R *= –0.316, *P = *1.85^e–24^).

### APOBEC3B-related gene enrichment analysis data

To further study how expression of the *APOBEC3B* gene affects tumorigenesis, we screened *APOBEC3B*-binding proteins and *APOBEC3B* expression-related genes, and carried out a series of pathway and function enrichment analyses. Through the STRING tool, we screened the top 50 *APOBEC3B* binding proteins based on text mining, experiments, databases, co-expression, Gene Fusion, and co-occurrence. Figure 5a shows the interaction network of these proteins. We then obtained the top 100 genes related to *APOBEC3B* expression based on all tumor expression data of TCGA using the GEPIA2 tool. As shown in Fig. 5b, the expression level of *APOBEC3B* was positively correlated with those for the genes encoding thymidine kinase 1 (TK1; *R* = 0.49), maternal embryonic leucine zipper kinase (MELK; *R* = 0.47), centrosomal protein 55 (CEP55; *R* = 0.46), minichromosome maintenance complex component 2 (MCM2; *R* = 0.46) and non-SMC condensin I complex subunit H (NCAPH; *R* = 0.45) (all *P <* 0.001). The corresponding heatmap data also showed that the expression level of *APOBEC3B* was positively correlated with the above five genes in most tumor types (Fig. 5c). The top 50 *APOBEC3B*-binding proteins and the top 100 *APOBEC3B* expression-related-genes were cross analyzed to obtain a common gene, namely that encoding karyopherin subunit alpha 2 (KPNA2; Fig. 5d).

To further clarify the function of *APOBEC3B* in tumors, we combined the top 50 *APOBEC3B*-binding proteins and the top 100 co-expression genes above for KEGG (www.kegg.jpkegg/kegg1.html) and GO enrichment analysis. KEGG data show that the terms ‘thermogenesis’, ‘non-alcoholic fatty liver disease’ and ‘oxidative phosphorylation’ might be involved in the mechanism of *APOBEC3B* affecting tumorigenesis. The GO enrichment analysis data showed that most of these genes are involved in or regulate the mitochondrial oxidative respiratory chain and its related pathways, such as the respiratory electron transport chain, ATP synthesis-coupled electron transport, mitochondrial ATP synthesis-coupled electron transport, and the mitochondrial respiratory chain among others. These data of KEGG and GO enrichment analysis have been visualized as a bubble plot (Fig. 5e).

## Discussion

Gene mutations are closely related to the occurrence of tumors, and the sources that mediate mutation are divided into exogenous and endogenous types [27, 28]. Endogenous mutation sources involve enzymes acting in the DNA repair system, and there are reports that the mutation mode of APOBEC cytidine deaminases might play a role in carcinogenic somatic mutations and eventually lead to genomic instability [29, 30]. The human APOBEC protein family has seven members and are important members of the innate immune system [31]. Thus, *APOBEC3B* had the highest expression in bone marrow among normal tissues in our study. It not only participates in innate immunity, but also participates in a variety of biological processes in different cells. In particular, the *APOBEC3B* protein with cytosine deaminase activity can lead to base substitutions in tumor-causing genes [32, 33]. Many publications have reported that *APOBEC3B* is closely related to the occurrence and development of a variety of tumors [10, 13, 14, 34]. However, there is still a lack of in-depth research on whether *APOBEC3B* can play a role in different tumors through molecular mechanisms. We searched the literature of major databases and failed to retrieve any pan-cancer analysis publications on *APOBEC3B* from the perspective of tumors as a whole. Therefore, based on the TCGA and CPTAC databases, we comprehensively analyzed the gene expression, gene changes and immune infiltration of the *APOBEC3B* gene in 33 different tumors.

*APOBEC3B* proved to be highly expressed in most tumor tissues, but weakly in a few types, such as COAD, LAML, READ, THCA, and THYM. To minimize errors caused by individual differences in tumor data, we downloaded the RNAseq pan-cancer data in level 3 HTseq-FPKM format from the TCGA project, selected paired sample data, and then analyzed the differential expression of *APOBEC3B* through the ‘ggplot2’ R package. The results obtained are basically consistent with Fig. 1a (see also Additional file 2: Figure. S1c). We also found that in some tumors, the expression of *APOBEC3B* was mainly positively correlated with the tumor’s pathological stage, but in CHOL and OV tumors, the expression of *APOBEC3B* was negatively correlated with pathological stage. These results indicate that the expression trend of *APOBEC3B* is different in different tumors, and it may play a different role in their progression.

In the patient survival analysis, we found that the relationship between *APOBEC3B* expression and survival obtained by GEPIA2 was basically the same for OS and DFS. In most tumors, a high expression level of *APOBEC3B* indicated a poor prognosis, such as ACC, KIRP, LGG, LIHC, THCA, and UVM (but not in CESC). To further confirm the reliability of survival analysis data, we used Kaplan–Meier plotter tool for progression-free survival (PFS) analysis of the above cancers with different prognoses. The results (Additional file 2: Figure. S2) showed that high expression levels of *APOBEC3B* in ACC, LGG, LIHC, THCA and UVM were associated with poor PFS prognosis. It was associated with the low expression of *APOBEC3B* in CESC, but it was not statistically significant in KIRP. Thus, the expression level of *APOBEC3B* is closely linked to the prognosis and survival of patients in some tumor cases, but in different ways. Therefore, a larger sample size is needed to test the role of *APOBEC3B* in the survival and prognosis of different types of cancers.

Gene alterations play important roles in tumorigenesis. We found that an *APOBEC3B* gene alteration was associated with patient prognosis and survival in SKCM, and the prognosis for the gene-altered group was significantly worse than that of the unaltered group. However, in other tumors with an *APOBEC3B* gene alteration, we did not find an impact of gene alterations on patient prognosis and survival. Because of the small number of altered group cases for most tumors, we think it is necessary to further expand the data sample for verification in the future. In addition, we also analyzed the relationship between the expression levels of *APOBEC3B* and tumor mutational burden (TMB) and microsatellite instability (MSI) in all TCGA tumors. MSI often reflects a defect in the DNA mismatch repair system and the TMB reflects cancer mutation quantity. [35, 36] Additional file 2: Figure S3 shows that the expression level of *APOBEC3B* was positively correlated with the TMB of ACC, BLCA, BRCA, CESC, COAD, LIHC, LUAD, OV, PRAD, PAAD, SKCM, THCA, and THYM. The expression level of *APOBEC3B* was also negatively correlated with the MSI of BRCA, HNSC, KIRC, LIHC, STAD, and TGCT. These findings deserve further analysis.

Cancer-associated fibroblasts are considered to be a form of mutant cells with negative epithelial, endothelial and leukocyte markers, with slender morphology not found in cancer cells [37]. They have a variety of functions, including matrix deposition and remodeling, extensive signal interaction with cancer cells, and cross-talking with invasive leukocytes [38]. Therefore, they are potential factors affecting cancer treatment strategies. CD8^+^ cytotoxic T lymphocytes are immune cells that can target cancer, and tumor-associated fibroblasts, macrophage type 2 cells and regulatory T cells can form an immune barrier to the anti-tumor immune response mediated by such cells [39]. Our study is the first to suggest an association between the expression of *APOBEC3B* and the level of immune cell infiltration of tumor-associated fibroblasts and CD8^+^ T cells in some tumors. In addition, we integrated gene information related to *APOBEC3B* binding or affecting *APOBEC3B* expression in all tumors via the STRING and GEPIA2 databases, and conducted a variety of interaction, correlation and enrichment analyses. In addition, we integrated gene information related to *APOBEC3B* binding or affecting *APOBEC3B* expression in all tumors through a variety of databases, and conducted a variety of interaction, correlation and enrichment analyses (Table S2). Finally, we found that the GO terms ‘ATP synthesis-coupled electron transport’, ‘thermogenesis’ and ‘oxidative phosphorylation’ might be involved in the pathogenesis of cancer. Moreover, we screened for the KPNA2 gene based on two databases, and found that its expression was highly positively correlated with the expression level of *APOBEC3B*. KPNA2 is a member of the nuclear transport protein family. In many studies, KPNA2 has been linked to tumorigenesis (such as non-small cell lung cancer, breast cancers, and epithelial ovarian cancer), [40–42] and the gene is highly expressed in many cancerous tissues, which is consistent with the expression of the *APOBEC3B* gene in this study. It has a cancer promoting effect in in vivo and in vitro experiments, and is often related to a poor prognosis for patients [43]. We hypothesize that there might be some mechanism connecting *APOBEC3B* and KPNA2 that promotes the occurrence and development of tumors, which needs to be further tested by in vivo and in vitro experiments in the future.

## Conclusions

Here we conducted pan-cancer analysis of *APOBEC3B,* explored the relationship between *APOBEC3B* expression levels and clinical prognosis, immune cell infiltration, TMB or MSI, as well as the mutation sites, interactive genes and involved pathways of the *APOBEC3B* gene. These findings indicate that APOBEC3B might have different biological functions in different cancer species. Their main significance lies in the need to take such differences into account when designing drugs targeted for individual genes (Additional file 3: Table S2).

## Supplementary Information

**Additional file 1**. Clinical prognostic significance of DNA methylation of APOBEC3B.

**Additional file 2**. An overview of apobec3b and the relationship between its expression and PFS or TMB.

**Additional file 3**. GO and KEGG enrichment analysis of APOBEC3B related genes.

## Data Availability

The datasets generated and analyzed during the current study are available in the TCGA repository, https://www.cancer.gov/about-nci/organization/ccg/research/structural-genomics/tcga.

## Abbreviations

ACC: Adrenocortical carcinoma
*APOBEC3B*: Apolipoprotein B mRNA editing enzyme catalytic subunit 3B
BLCA: Bladder urothelial carcinomas
BP: Biological process
CC: Cellular component
CEP55: Centrosomal protein
CESE: Cervical squamous cell carcinomas and endocervical adenocarcinomas
CHOL: Cholangiocarcinomas
CNA: Copy number alteration
COAD: Colon adenocarcinomas
CPTAC: Clinical proteomic tumor analysis consortium
DBLC: Lymphoid neoplasm diffuse large B-cell lymphomas
DFS: Disease-free survival
ESCA: Esophageal carcinomas
GBM: Glioblastoma multiforme
GEPIA: Gene expression profiling interactive analysis
GO: Gene ontology
GTE: Genotype-tissue expression
HNSC: Head and neck squamous carcinomas
HPV: Human papilloma virus
KEGG: Kyoto encyclopedia of genes and genomes
KICH: Kidney chromophobes
KIRC: Kidney renal clear cell carcinomas
KIRP: Kidney renal papillary cell carcinomas
KPNA2: Karyopherin subunit alpha 2
LAML: Acute myeloid leukemia
LGG: Brain lower grade gliomas
LIHC: Liver hepatocellular carcinomas
LUAD: Lung adenocarcinomas
LUSC: Lung squamous cell carcinomas
MCM2: Minichromosome maintenance complex component 2
MELK: Maternal embryonic leucine zipper kinase
MESO: Mesotheliomas
MF: Molecular function
MSI: Microsatellite instability
NCAPH: Non-SMC condensin I complex subunit H
OV: Ovarian cancers
PAAD: Pancreatic adenocarcinomas
PCPG: Pheochromocytomas and paragangliomas
PFS: Progression-free survival
PRAD: Prostate adenocarcinomas
READ: Rectum adenocarcinoma
SKCM: Skin cutaneous melanomas
STAD: Stomach adenocarcinomas
TCGA: The cancer genome atlas
TGCT: Testicular germ cell tumors
THCA: Thyroid carcinomas
THYM: Thymomas
TIMER: Tumor immune estimation resource
TK1: Thymidine kinase 1
TMB: Tumor mutational burden. TPM: transcripts per million reads
UCEC: Uterine corpus endometrial carcinomas
UCS: Uterine carcinosarcomas
UVM: Uveal melanomas
