# Supplementary material for: Multiple roles of apolipoprotein B mRNA editing enzyme catalytic subunit 3B (APOBEC3B) in human tumors: a pan-cancer analysis
Source: BMC Bioinformatics. 2022 Aug 2;23:312. doi: 10.1186/s12859-022-04862-0 (PMC9344753; doi:10.1186/s12859-022-04862-0)
Supplement: Supplementary file 2 — Additional file 2. An overview of apobec3b and the relationship between its expression and PFS or TMB. [file 12859_2022_4862_MOESM2_ESM.docx]

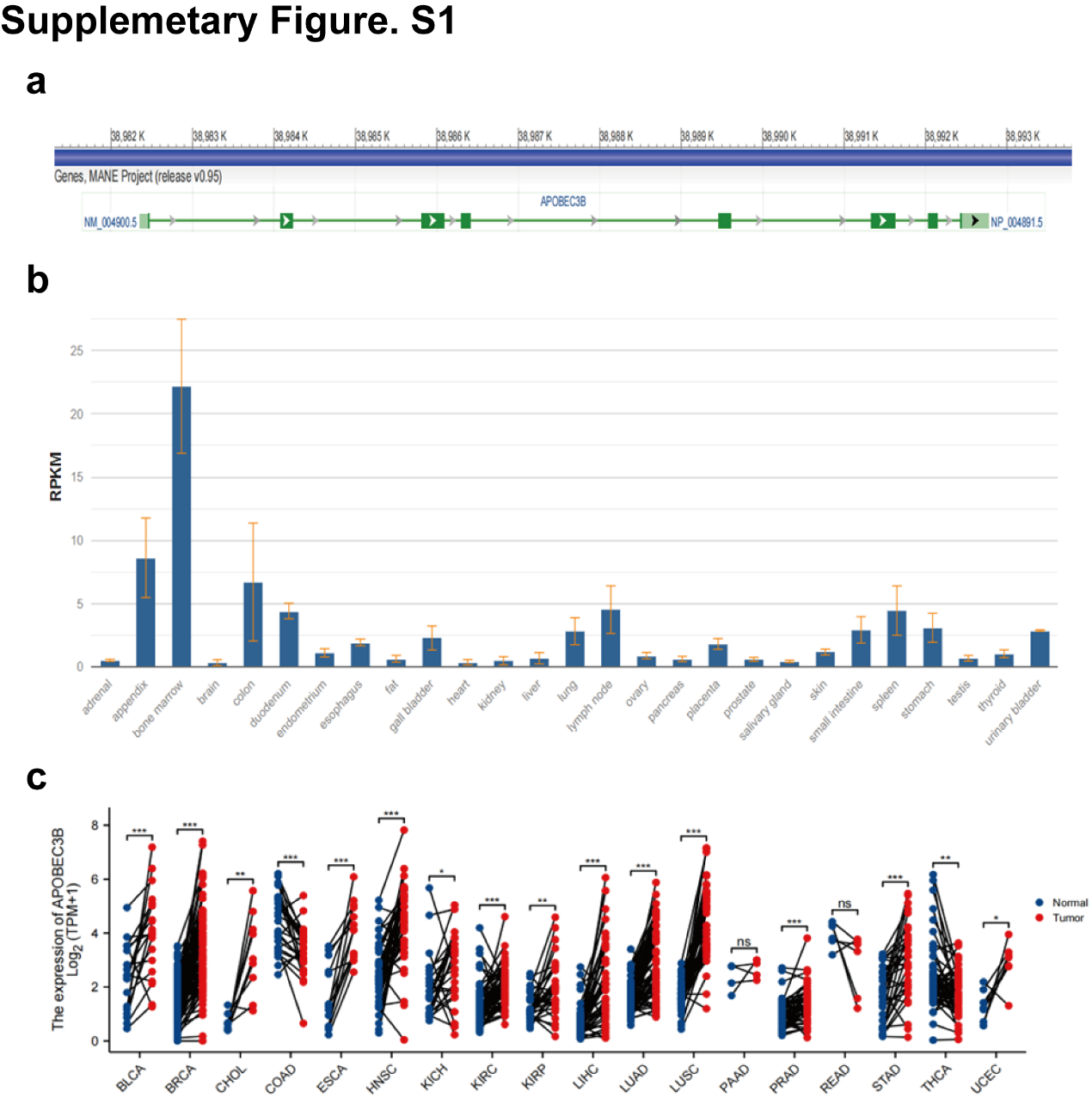


Supplementary Figure S1. **Overview and Expression of APOBEC3B**

a. Overview of *APOBEC3B* (NM_004900.4 for mRNA and NP_004891.4 for protein)

b. The mRNA expression of APOBEC*3B* in normal tissues.

c. Expression of *APOBEC3B* in paired tumor and adjacent normal tissues.


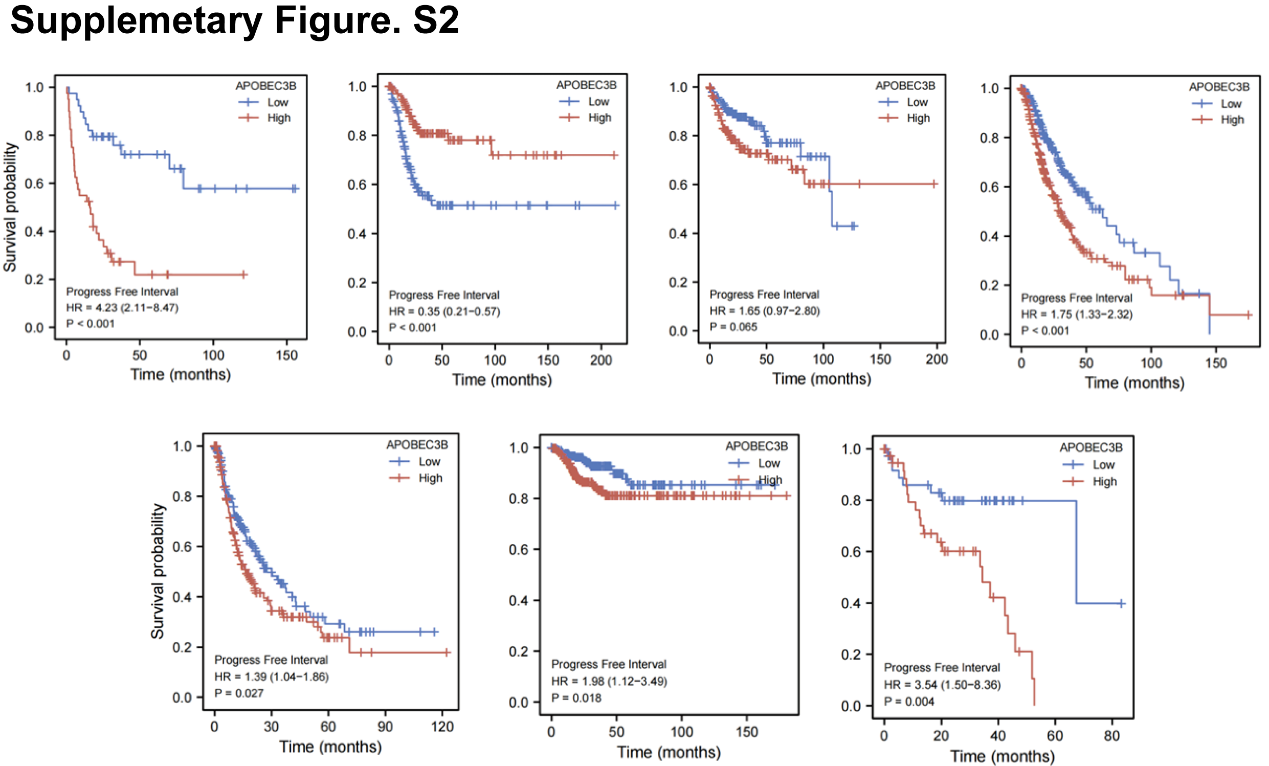


Supplementary Figure S2. **High expression levels of APOBEC3B were associated with poor PFS prognosis in ACC, LGG, LIHC, THCA and UVM.**


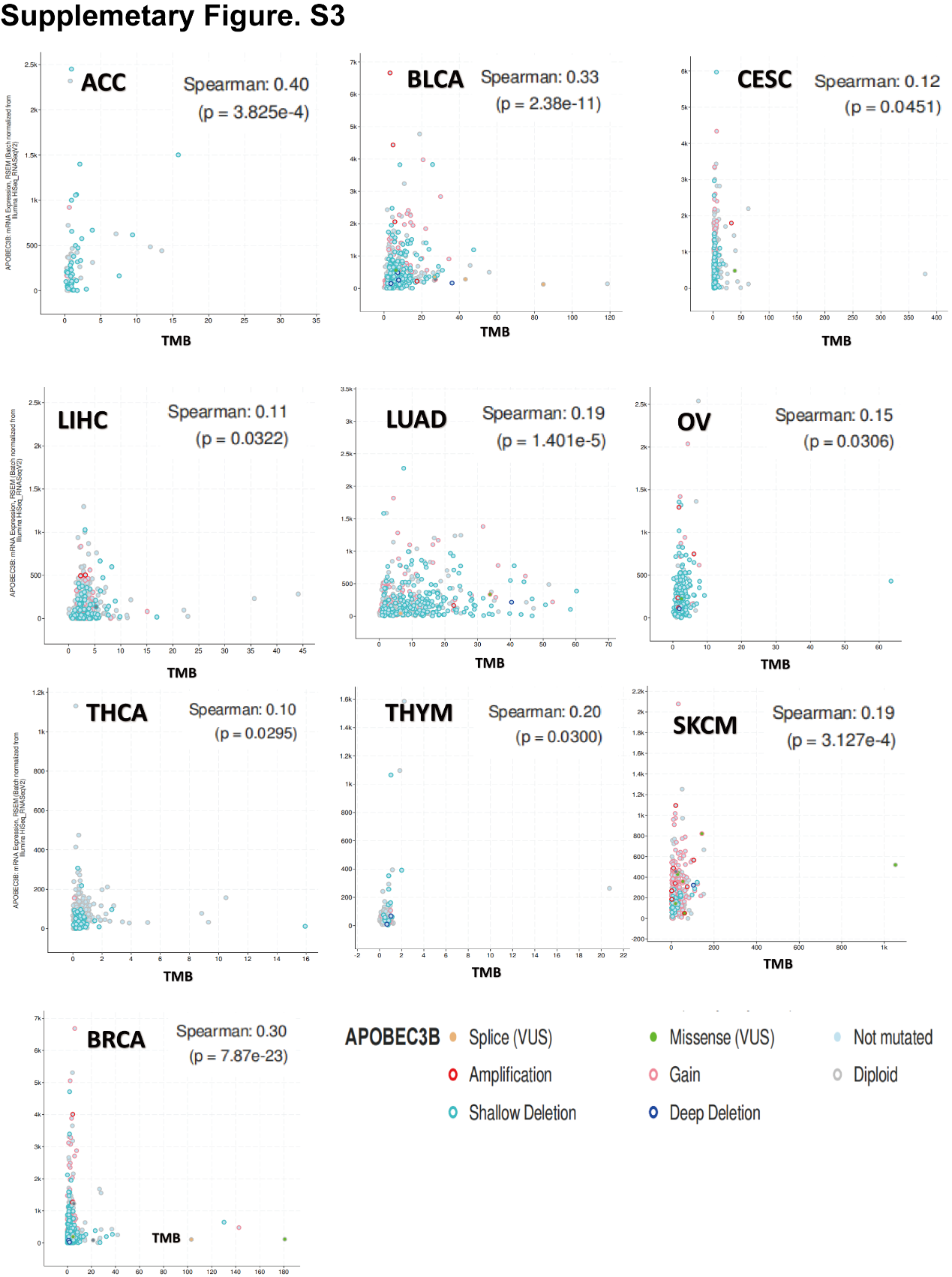


Supplementary Figure S3. **The expression level of APOBEC3B was positively correlated with the TMB in ACC, BLCA, BRCA, CESC, COAD, LIHC, LUAD, OV, PRAD, PAAD, SKCM, THCA, and THYM.**
