# Supplementary material for: Multiple roles of apolipoprotein B mRNA editing enzyme catalytic subunit 3B (APOBEC3B) in human tumors: a pan-cancer analysis
Source: BMC Bioinformatics. 2022 Aug 2;23:312. doi: 10.1186/s12859-022-04862-0 (PMC9344753; doi:10.1186/s12859-022-04862-0)
Supplement: Supplementary file 3 — Additional file 3. GO and KEGG enrichment analysis of APOBEC3B related genes. [file 12859_2022_4862_MOESM3_ESM.docx]

**Table S2. GO and KEGG enrichment analysis of APOBEC3B related genes.**

| ONTOLOGY | ID | Description | GeneRatio | BgRatio | pvalue | p.adjust | qvalue |
| --- | --- | --- | --- | --- | --- | --- | --- |
| BP | GO:0042773 | ATP synthesis coupled electron transport | 27/139 | 98/18670 | 1.60e-35 | 2.38e-32 | 1.75e-32 |
| BP | GO:0042775 | mitochondrial ATP synthesis coupled electron transport | 26/139 | 97/18670 | 7.23e-34 | 5.40e-31 | 3.96e-31 |
| BP | GO:0022904 | respiratory electron transport chain | 27/139 | 117/18670 | 3.44e-33 | 1.71e-30 | 1.26e-30 |
| BP | GO:0006119 | oxidative phosphorylation | 27/139 | 145/18670 | 1.88e-30 | 7.02e-28 | 5.15e-28 |
| BP | GO:0045333 | cellular respiration | 28/139 | 193/18670 | 2.27e-28 | 6.78e-26 | 4.98e-26 |
| CC | GO:0098803 | respiratory chain complex | 22/142 | 85/19717 | 1.16e-28 | 2.58e-26 | 1.88e-26 |
| CC | GO:0070469 | respiratory chain | 22/142 | 100/19717 | 6.18e-27 | 6.86e-25 | 5.01e-25 |
| CC | GO:0005746 | mitochondrial respiratory chain | 21/142 | 88/19717 | 1.45e-26 | 1.07e-24 | 7.85e-25 |
| CC | GO:1990204 | oxidoreductase complex | 20/142 | 112/19717 | 1.32e-22 | 7.34e-21 | 5.36e-21 |
| CC | GO:0098800 | inner mitochondrial membrane protein complex | 21/142 | 135/19717 | 2.29e-22 | 1.02e-20 | 7.42e-21 |
| MF | GO:0003954 | NADH dehydrogenase activity | 11/139 | 46/17697 | 4.96e-14 | 4.30e-12 | 3.27e-12 |
| MF | GO:0008137 | NADH dehydrogenase (ubiquinone) activity | 11/139 | 46/17697 | 4.96e-14 | 4.30e-12 | 3.27e-12 |
| MF | GO:0050136 | NADH dehydrogenase (quinone) activity | 11/139 | 46/17697 | 4.96e-14 | 4.30e-12 | 3.27e-12 |
| MF | GO:0009055 | electron transfer activity | 14/139 | 114/17697 | 2.80e-13 | 1.82e-11 | 1.38e-11 |
| MF | GO:0016655 | oxidoreductase activity, acting on NAD(P)H, quinone or similar compound as acceptor | 11/139 | 60/17697 | 1.16e-12 | 5.26e-11 | 4.00e-11 |
| KEGG | hsa00190 | Oxidative phosphorylation | 25/86 | 133/8076 | 3.39e-25 | 2.74e-23 | 1.96e-23 |
| KEGG | hsa04932 | Non-alcoholic fatty liver disease | 25/86 | 150/8076 | 8.07e-24 | 3.27e-22 | 2.34e-22 |
| KEGG | hsa04714 | Thermogenesis | 27/86 | 231/8076 | 1.68e-21 | 4.53e-20 | 3.24e-20 |
| KEGG | hsa05012 | Parkinson disease | 27/86 | 249/8076 | 1.26e-20 | 2.55e-19 | 1.82e-19 |
| KEGG | hsa05020 | Prion disease | 27/86 | 273/8076 | 1.45e-19 | 2.34e-18 | 1.68e-18 |
